# Chemical- and Mechanical-Induced Lubrication Mechanisms during Hot Rolling of Titanium Alloys Using a Mixed Graphene-Incorporating Lubricant

**DOI:** 10.3390/nano10040665

**Published:** 2020-04-02

**Authors:** Ning Kong, Jiaming Zhang, Jie Zhang, Hongbo Li, Boyu Wei, Dongshan Li, Hongtao Zhu

**Affiliations:** 1 School of Mechanical Engineering, University of Science and Technology Beijing, Beijing 100083, China; zjm1018774382@163.com (J.Z.); ZhangJie@ustb.edu.cn (J.Z.); lihongbo@ustb.edu.cn (H.L.); g20188554@xs.ustb.edu.cn (B.W.); ononlds@126.com (D.L.); 2 State Key Laboratory of Solid Lubrication, Lanzhou Institute of Chemical Physics, Chinese Academy of Science, Lanzhou 730000, China; 3 Faculty of Engineering and Information Sciences, University of Wollongong, Wollongong, NSW 2522, Australia; hongtao@uow.edu.au

**Keywords:** titanium alloy, tribological properties, hot rolling, mixed graphene-incorporating lubricant, lubrication mechanism

## Abstract

Hot rolling of titanium alloy currently is carried out without lubrication because of the surface defects. In order to explore an effective lubrication scheme to reduce friction and wear during hot rolling of titanium alloy, a mixed graphene-incorporating lubricant has been proposed to study its lubrication performance and mechanism. The tribological experiments were carried out by ball-disk friction and wear tester under hot-rolling parameters. Scanning electron microscopy (SEM), X-ray energy spectrum analyzer (EDS), X-ray powder diffractometer (XRD) and Raman analysis were used to analyse the surface and cross-section of the wear marks on the samples after the tribological experiments. The results show that the friction coefficient decreases up to about 35% compared with tests under dry and lubricated conditions. The surface quality of the wear marks is improved significantly after applying the proposed lubricant. The graphene which is embedded in the phosphate film can be effectively applied as a lubricating material to strengthen the lubricating film with less combustion loss at high temperatures. A chemical- and mechanical-induced lubrication mechanism for the hot rolling of titanium sheets has been proposed due to the synergistic lubrication effect of the graphene, ZrO_2_ nano particles and phosphate. It is of great significance and potential value to apply this proposed lubricant as an effective way to reduce the wear, friction and oxidation during the hot-rolling process of titanium alloy.

## 1. Introduction

**Figure 1:**
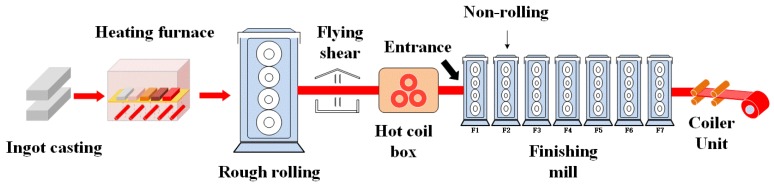
Hot-rolling process of titanium alloy sheets.

Titanium alloys present various advantages, such as high specific strength, good toughness, corrosion resistance and creep resistance. They are considered to be one of the materials with broad application prospects. Titanium alloy products have been widely used in fields such as aerospace, vehicle manufacturing and biomedicine [1,2]. Rolling is an important way to effectively produce titanium alloy through plastic deformation. According to statistics, in the production of titanium alloy, the ton number of rolled titanium alloy accounts for more than half of the total production number of titanium alloy [3,4]. Hot rolling is a very critical stage in the production of titanium alloy. The surface quality of titanium alloy after hot rolling directly affects the performance of the final product [5]. The process of hot rolling with titanium alloy is shown in Figure 1. During the hot-rolling process, the titanium alloys obtained a very marked crystallographic texture [6]. Different crystallographic textures of titanium and its alloys present different tribological performance. The wear rate also depends on the crystallographic structures of titanium alloys [7,8]. Hexagonal close packed (HCP) structure presents better abrasive wear resistance than the body-centered structures [6].This is why the hot-rolling process is done in a crosswise way. At present, the hot-rolling process of titanium alloy is carried out through a hot continuous rolling mill in industrial production without lubrication. However, under the action of high temperature and high pressure, the surface defects such as pits, indentations and scratches often appear on the surface of hot-rolled titanium alloy, as well as warping and flatness defects. These defects may lead to a product failure due to the surface quality requirements of titanium alloy [9,10,11]. It also decreases the life of the work rolls for the hot-rolling mills. It is suggested that the improvement of the contact conditions between the roll and titanium surface is a way to reduce the unqualified rate for the hot-rolled titanium product.

Suitable lubricant is a direct and effective method to improve the surface contact state. It is of great significance to optimize the rolling process of titanium alloy. Since the 1970s, a number of research works have been focused on the development of lubrication such as iodine and olefinic lubricants for the titanium. The friction reduction performance is attribute to the beneficial transfer films on the counterface and permanent changes in the nature of the lubricating fluid [6]. However, there is no suitable lubricant for hot continuous rolling of titanium alloys because the rolling speed is fast and the surface properties of titanium alloys are relatively active. The application of common water-based lubricants for cooling and lubrication will lead to a material absorption with large amounts of hydrogen elements, which is responsible for the hydrogen embrittlement. The failure mechanism is the formation of brittle titanium hydride by titanium and hydrogen at high temperature. The density of titanium hydride is less than the lattice density of metal and its volume is about 23% larger than that of the matrix phase Moreover, with the temperature increases, the hydrogen atom migration accelerates, the solution limit increases, and the formation of titanium hydride phase increases as well, which reduces the plasticity of titanium alloy and makes the surface of the titanium alloy more brittle. It will then affect the subsequent use and further deformation [12,13]. Therefore, it is very important to propose a sustainable lubrication with excellent lubrication performance for the hot rolling of titanium alloy [14,15]. The hot-rolling temperature of titanium alloys is generally over 60 °C, so the selection of high-temperature lubrication materials is a very important issue [16]. It has been pointed out in literature that the mixture of two or more solid lubricants is one of the promising strategies for developing high-temperature solid lubricants [17,18]. Based on this point of view, it is believed that a variety of mixed lubricating additives with different functions is able to achieve better lubrication performance. Graphene is a popular two-dimensional material and it has attracted great interest because of its excellent physical, chemical and mechanical properties [19,20,21]. Wang [20] has studied the tribological properties of graphene lubricant additives under different contact modes. The results show that graphene additives obviously improve the wear resistance and friction reduction of the original lubricant. Graphene can enhance and protect the friction film, and contribute to the formation of iron oxide friction film. In addition, the shearing property of graphene is able to effectively reduce the friction coefficient, and then improve its lubrication performance. Qi [22] used a multi-functional reciprocating friction and wear tester to investigate the effects of load and frequency on the tribological properties of graphene as a liquid paraffin additive. The results show that the friction coefficient and wear rate decrease when graphene is added at different loads with same frequency. Phosphate is a kind of heat-resistant material developed on the basis of cement, refractory and ceramic materials. It can be used to bond cermets, as well as to establish binders for composites, refractories, insulation materials and coatings [23,24,25]. Le [26] made use of the high-temperature resistance of phosphate binder and substituted acid phosphate binder for silicate binder to prepare alumina fiber membranes for high-temperature hot gas filtration and wastewater treatment. The flexural strength and creep resistance of alumina fiber membranes at high temperature have been greatly improved. Nano-materials also present the characteristics of large specific surface area, high diffusivity, easy sintering and low melting point [27,28]. These properties of nano-materials can also be used as a lubrication additive to reduce friction and wear resistance. Its lubrication mechanisms, including rolling/ball-bearing effect, protective film/tribofilm, mending effect, polishing effect, synergistic effect and third-body effect have been proposed to explain the lubrication enhancement of the nano lubricants [29]. Laura [30] studied the effect of nanoparticles on friction and wear. This indicates that nanomaterials can effectively reduce the wear degree and friction coefficient. Bao [31] studied the effect of nano-SiO_2_ on the surface morphology, microstructure and oxide scale of rolled strip by a hot-rolling test. The results show that when the mass fraction of Nano-SiO_2_ is less than 0.5 wt %, the surface morphology becomes smooth. The thickness of oxide scale decreases from 15 to 8 microns, and the grain size of surface microstructures is refined. The nano-silica presents good lubricity and is able to improve surface quality. It is due to the micro-rolling, polishing and self-repairing function of nano-silica on the strip surface. Nano zirconia is an important structural and functional material, which presents excellent properties in strength, toughness, corrosion resistance, wear resistance and thermal fatigue. Nano zirconia also shows a small size effect, quantum effect, surface effect and interface effect. It possesses many advantages that traditional solid lubricant additives cannot achieve. Compared with other oxide nano powder, nano zirconia shows lower thermal conductivity and thermal shock resistance, so it is more suitable for the hot-rolling environment. Nano zirconia also can reduce the fluctuation of friction coefficient and keep the whole deformation process more stable.

In terms of base fluids, water is regarded as the promising base lubricant for application because of its environmental protection and recyclability [32,33]. It was mentioned above that lubricants containing water may cause hydrogen embrittlement, resulting in deterioration of mechanical and processing properties of materials. However, it is also mentioned in literature [12,34] that proper hydrogen can improve the plasticity of titanium alloys. For example, hot hydrogen treatment technology is used in the forming process. This technology is able to achieve an optimal structure for the titanium and hydrogen elements by the hydrogen causing plasticity and hydrogen causing a phase transformation mechanism. Therefore, it improves the processing performance and efficiency of titanium alloys. The hydrogen is a stable element of the beta phase, which can increase the stability of the beta phase in titanium alloys. In addition, hydrogen can weaken the bonding between metal atoms, reduce the internal bonding energy of metal, soften the metal region and promote the appearance of dislocation. Cornelio [35] studied the application of carbon nanotubes as oil and water additives. The tribological properties of carboxylic acid modified functional nanotubes (single-wall and multi-wall) with different concentrations (0.01%, 0.05%) as lubricant additives were studied on a double-disk friction and wear tester. The results show that the friction coefficient and wear rate of the two systems (oil and water) decrease with the existence of carbon nanotubes. This indicates that water-based lubricants may achieve similar lubrication effects as oil-based lubricants. It is also of great significance and potential to study water-based lubricants as alternatives to oil-based lubricants [32,36].

At present, the development or selection of lubricants is still an industrial challenge for the hot rolling of titanium alloys sheet. Due to the higher surface quality requirement, the hot-rolling lubricant for titanium alloy is urgently needed so as to increase the strip yield. However, the corresponding lubricant, surface property, and lubrication mechanism during hot rolling of titanium alloys at high temperatures have not been systematically developed. In this work, a mixed graphene-incorporating lubricant is proposed for the hot rolling of titanium alloy sheets. The tribological experimental study has been carried out based on the hot-rolling parameters of titanium alloy. By means of scanning electron microscopy (SEM, TESCAN MIRA3, Brno, Czech), an energy-dispersion spectrometer (EDS, Bruker QUANTAX, Billerica, MA, USA), X-ray diffraction (XRD, PANalytical X’pert-PRO X-ray goniometer, Eindhoven, the Netherlands) and Raman spectroscopy (Renishaw-inVia, London, UK) characterization methods, the high temperature lubrication performance of the lubricant has been evaluated through the analysis of the morphology and the chemical products generated in the wear mark. The chemical- and mechanism-induced lubrication mechanism has been explored as well. It is of great significance and potential value to guide the development of the lubricant for the hot rolling of titanium alloy.

## 2. Materials and Methods

### 2.1. Preparation of Mixed Graphene-Incorporating Water-Based Lubricant

**Figure 2:**
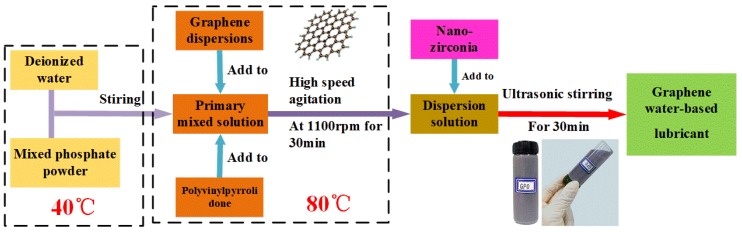
Schematic diagram of preparation method of proposed mixed graphene-incorporating lubricant.

In order to achieve an outstanding lubricating performance for high-temperature applications, the proposed lubricant requires high stability, wide temperature range and a long service life with good antiwear and friction-reduction performance. A mixture lubrication is an outstanding candidate for the proposed lubricant. The mixed graphene-incorporating water-based lubricant proposed in the experiment is composed of graphene, phosphate, ZrO_2_ nano-powder, polyvinylpyrrolidone and balanced water. The percentage of its constituent content is shown in Table 1. The phosphates include sodium tripolyphosphate, sodium pyrophosphate and potassium dihydrogen phosphate, each of which accounts for 3%. The detailed preparation process of the lubricant is described as follows. The graphene water dispersion solution is added to the weighted deionized water. Other components are then added to the mixed solution. Finally, the lubricant is obtained after ultrasonic dispersion in the water bath. The preparation of lubricants is shown in Figure 2.

### 2.2. Materials

GCr15 steel balls and Ti-0.3Mo-0.8Ni alloy have been used in this experiment. The chemical compositions of the two materials are listed in Table 2 and Table 3. The ball is 10 mm in diameter and with a hardness around 60 HRC. The original roughness of the ball is about 0.15–0.2 μm. The disks have been machined to 25 mm in diameter and 6 mm in thickness. The surface of titanium alloy is polished to a mirror state. Then the samples are cleaned with acetone solution and degassed with anhydrous ethanol solution.

### 2.3. Tribological Test and Characterization

**Figure 3:**
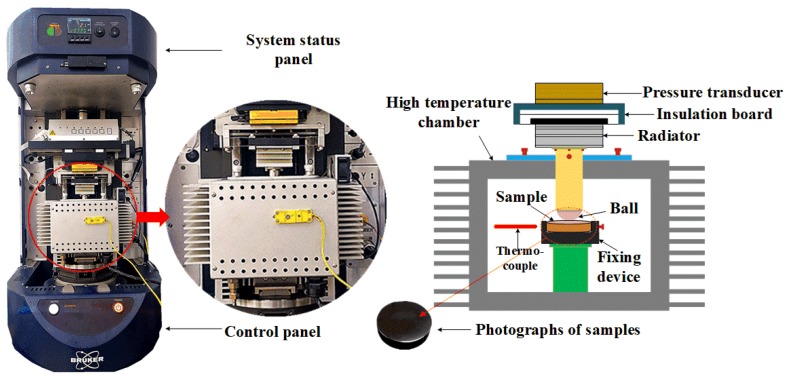
Appearance and principle charts of friction and wear testing machine used in the tribological tests.

The UMT-2 multifunctional friction and wear tester is mainly composed of a digital control module, loading module, temperature control module and reciprocating motion module. This friction and wear-testing machine is characterized by independent high-temperature module components, built-in high-sensitivity thermocouple and temperature control accuracy with 0.01 °C. A variety of standard and conventional tribological tests can be carried out for various materials to obtain load, friction coefficient and displacement at the same time for the tests. In order to simulate the real contact pressure, Hertz contact stress theory is applied to calculate the normal pressure for the tribotests. The pressure between the roll and workpiece on the first and 6th stands are 823.35 MPa and 779.56 MPa. The required experimental load can be calculated as 11.42 N and 9.69 N. The normal pressure is close to the rolling pressure. The tribological tests were carried out on a UMT-2 tribometer under dry friction and lubricated conditions in order to evaluate the lubrication performance of the proposed lubricant. The unlubricated state is not easy to be appropriately controlled. The tribotests have been repeated in order to obtain reasonable results. For the application, it is a way to study why there are always surface defects happening during the industrial hot-rolling process and the appearance of the defects in a micro view. It is also helpful to verify the meaning of the lubrication development for the hot rolling of titanium strips from a technology point of view. The synergistic actions of different lubricant components in a mixed state can be deeply investigated from both experimental and theory efforts. The coefficient of friction (COF) with titanium alloys can be achieved from the experiments. Figure 3 shows the configuration of the UMT-2 testing system. Friction and normal force are measured by sensors on the top of the upper sample during the experiment. The coefficient of friction in the experiment is calculated by computer software. During the experiment, the upper sample remains stationary while the lower sample moves in a reciprocating straight line. The normal force is applied mechanically to the upper specimen in a direction that is vertical to the motion direction with a test presetting frequency and stroke.

This work is carried out by the ball-on-disc tribological tests in order to prove the efficiency of the proposed lubricant. Four tribotests have been carried out in order to simulate the hot-rolling processes under dry and lubricated conditions for stands 1 and 6 of the finishing mill. In order to study the lubrication mechanism of the mixed graphene-incorporating water-based lubricant, two groups of comparative experiments were conducted under both dry and lubrication conditions for each stand of finishing mill. The determination of experimental conditions is based on the equivalent calculation of industrial production parameters (finishing mill of stands 1 and 6) during hot rolling of titanium alloys. The two detailed experimental conditions are shown in Table 4 below.

After experiments, the coefficient of friction was recorded automatically. SEM was used to study the detailed microstructure in the part of surface, cross-section and wear track. Chemical analysis was conducted by means of EDS and XRD.

## 3. Results and Discussion

### 3.1. Coefficient of Friction

**Figure 4:**
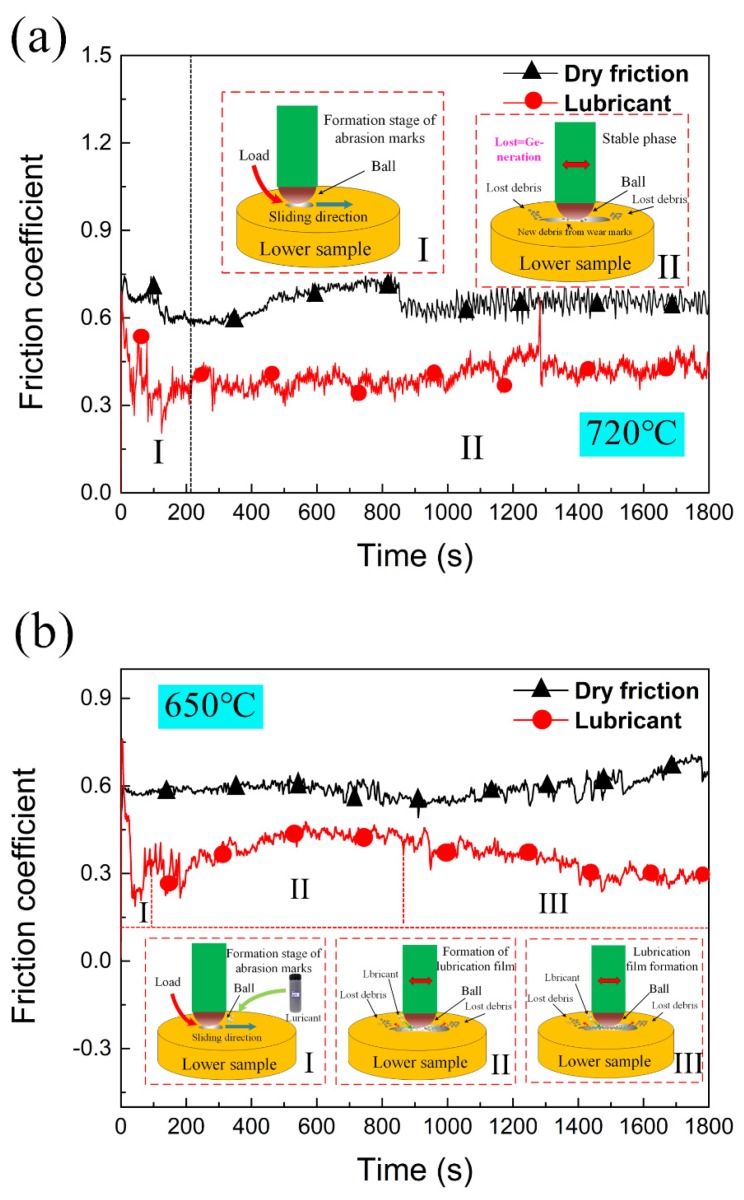
Coefficient of friction (COF) of Ti-0.3Mo-0.8Ni under (**a**) Condition 1 (720 °C/11.42 N/4.50 Hz) and (**b**) Condition 2 (650 °C/9.69 N/12.62 Hz).

Figure 4 shows the coefficient of friction (COF) curves under dry and lubricated friction conditions with different contact parameters. Figure 4a,b show the experimental results under condition 1 and condition 2 respectively. The abscissa represents time and the ordinate represents friction coefficient. The friction coefficient decreases markedly after using lubricant. In addition, a schematic diagram of the friction process under dry friction and lubricated conditions are shown in Figure 4a,b. The average COF under an unlubricated condition ranges from 0.57 to 0.75 under condition 1, compared with 0.3–0.45 after using lubricant. In condition 2, the average COF under unlubricated condition ranges from 0.55 to 0.70, while the COF under lubricated condition is stable at 0.25–0.45. The COF under condition 1 is generally higher than that of condition 2, and the COF measured after using lubricant is lower than that of measured unlubricated. The fluctuation of each curve obviously shows different contact states on the surface of the friction pair during the tribological process.

The trend of whole friction process can be analyzed by the schematic diagram of friction coefficient evolution obtained from experimental condition 1. The evolution process of COF obtained by the tribological tests follows a similar tendency with two stages. The first stage is the initial stage of friction. This is the stage with large variation of COF, which is characterized by the sudden increase of COF and slow decrease of COF in the beginning of the experiment. The measured COF reaches about 1.2 in a short time, and then slowly decreases to around 0.6. After that, the friction coefficient shows a relatively stable trend in the second stage and fluctuates slightly until the end of the friction experiment. The same evolution trend of friction coefficient can be seen from the curve of COF obtained under experimental condition 2 in Figure 4b. After a number of tribological tests and the SEM investigation in the following section, the reason for the first phase occurrence is that the upper sample adheres to the lower sample during the high-temperature loading process. Before the sliding track of the lower specimen is formed, the friction force is responsible for the plastic deformation. With the formation of wear track, the plastic deformation rate decreases, which leads to a downward trend of the friction coefficient. The average friction coefficient in this stage is higher than that in the next stage [37]. In the second stage, due to the generation of debris and the stable friction process, the change of friction coefficient in this stage presents a relatively stable trend. At the same time, the movement of wear debris in the wear scar forms an isolation layer, which transforms part of the sliding friction into rolling friction. It is able to reduce the friction coefficient.

Under lubricated conditions, the evolution of friction coefficient curve can be divided into three stages under a lubricated condition in Figure 4b. The first stage is generally consistent with that of dry friction. The plastic deformation force at the initial stage is provided by friction force, the friction coefficient increases to 0.9 firstly and then decreases slowly. The difference is in the second and third stages. After using lubricant, the friction coefficient of the second stage increases slightly. The reason is that the addition of lubricant leads to an increase of debris on the surface of friction pairs. In the third stage, the friction coefficient shows a downward trend. The friction state is stable at this time, and a relatively stable and compact isolation layer is formed on the surface of the friction pair. This trend continues until the end of the experiment, which shows that the isolation layer formed by this lubricant is not easily destroyed. It also proves that there is a synergistic effect among the components in the proposed lubricant. By comparing the dry friction condition, the friction coefficient after applying the mixed graphene-incorporating lubricant is always lower with a more stable tendency. This shows that the use of lubricant is helpful to improving the anti-friction and anti-wear performance in the whole tribological process.

Table 5 shows the average friction coefficients of both dry and lubrication conditions under the experimental conditions of 1 and 2. After applying lubrication, the average friction coefficient decreases by 30.27% (condition 1) and 35.22% (condition 2), respectively. The wear volumes of titanium discs are 0.23 mm^3^ and 0.14 mm^3^ in dry and lubricated tests under condition 1. While the wear volumes are 0.18 mm^3^ and 0.09 mm^3^ in dry and lubricated tests under condition 2. This proves that the proposed lubricant plays a critical role in reducing the friction coefficient and wear volume during the hot rolling of titanium alloy sheets.

### 3.2. Surface Morphology

**Figure 5:**
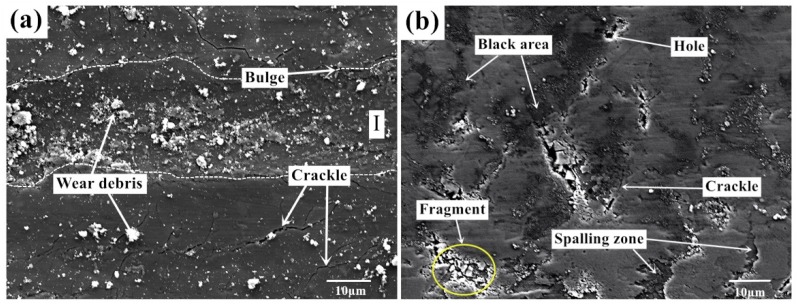
Morphology of scratched surface with dry friction under (**a**) Conditions 1 (720 °C/11.42 N/4.50 Hz) and (**b**) Conditions 2 (650 °C/9.69 N/12.62 Hz), 5000×.

Figure 5 shows the surface morphology of Ti-0.3mo-0.8Ni alloy with two experimental processes under a dry friction condition. The wear surface of Ti-0.3Mo-0.8Ni titanium alloy presents plastic deformation, tear marks and plough marks parallel to the sliding direction under the action of elevated temperature and high contact stress, which are typically defined as adhesion wear and abrasive wear. A number of large fragments can be seen on the worn surface as well. In the adhesion and sliding processes, strong ion bonds and other covalent bonds form between the micro-convex bodies of friction pairs, which are sheared under the action of contact load. Thus, larger size debris form in this process. Large debris is easily detached from the surface, which is not conducive to the formation of stable friction layer. A large amount of granular substances and debris accumulate on the surface of wear marks. It can be inferred that the debris comes from the oxide scale of titanium. There are some holes on the worn surface as well. It can be judged comprehensively that the plough formed in the sliding process of the titanium alloy introduces the metal plastic deformation. As the friction continues, the accumulated part is flattened again. After repeated plastic deformation, cracks begin to appear and extend into the alloy substrate. The flake peeling and fatigue damage then occur on the surface. According to the characteristics on the worn surface, the wear mechanism of Ti-0.3mo-0.8Ni alloy during the hot-rolling process is concluded to the combination effect of abrasive wear, adhesive wear and oxidation wear.

**Figure 6:**
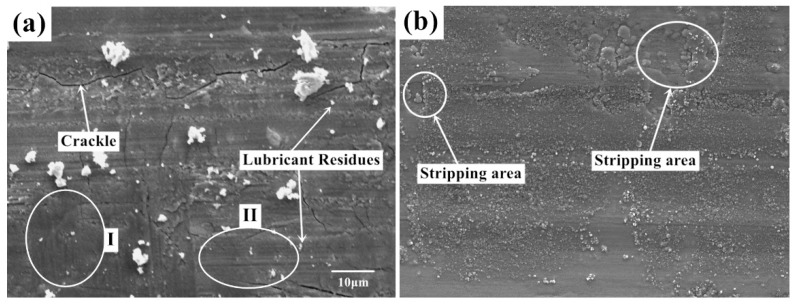
Morphology of scratched surface with lubrication under (**a**) Conditions 1 (720 °C/11.42 N/4.50 Hz) and (**b**) Conditions 2 (650 °C/9.69 N/12.62 Hz), 5000×.

Figure 6 shows the micro-morphology of the worn surface with lubrication under two experimental conditions. Figure 6a,b are the surface morphology in condition 1 (720 °C /11.42 N/4.50 Hz) and 2 (650 °C /9.69 N/12.62 Hz). The surface quality of the sample has been greatly improved after using lubricant. Compared with the samples obtained from dry friction, the lubricated surface is smoother and the surface defects are greatly reduced. The surface cracks, holes, and peeling debris are significantly decreased. The plough on the worn surface is no longer obvious. In Figure 6a, there are still some small particles with different sizes on the specimen surface. Some cracks can also be observed on the worn surface. Furthermore, there are almost no wear marks parallel to the friction direction at Mark I compared with the morphology at Mark II, and the structure is relatively uniform and dense. In Figure 6b, the surface obtained with lubricant also shows a typical characteristics of abrasive wear-strip wear marks. There are also different sizes of debris mounted on the surface without obvious tears and raised marks. The wear form of the wear marks are abrasive wear and oxidation wear under this contact condition. This shows that the use of lubricants greatly improves the severe wear between friction pairs. In Figure 6a, under the action of friction movement, the lubricant materials and contact pairs form a protective film near position I. The lubrication film in this area is continuous and compact with deformation patterns. In Figure 6b, a relatively large area of uniform coverage of lubrication film can be found. The protective film forms an isolation layer at the friction interface, which prevents the uneven contact between friction pairs. The leading wear-type transforms to the abrasive wear and oxidation wear.

**Figure 7:**
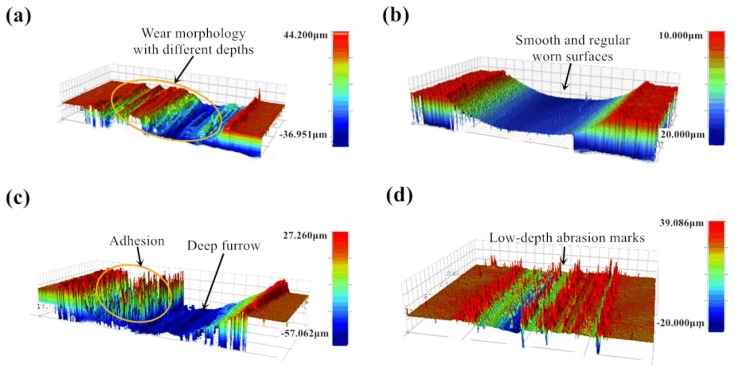
3-D topographies of the sample under Conditions 1 (720°C/11.42 N/4.50 Hz) with (**a**) dry friction; (**b**) after using lubricant; Condition 2 (650 °C/9.69 N/12.62 Hz) with (**c**) dry friction and (**d**) after using lubricant.

In order to verify the effect of proposed lubricant on the worn track from a macroscopic point of view, three-dimensional morphologies were measured under dry friction and lubricated conditions. Figure 7 a–d show the three-dimensional topography of the tests. The morphology and surface quality of the friction interface of the sample were greatly improved after using lubricant.

The width of wear marks with and without using lubricant does not change much, but the wear depth is quite different. The wear depth of the sample with lubricant is lower than that of without lubricant. From Figure 7a,c, the wear marks produced under dry friction condition are irregular with different distribution and depths. There are large distribution variation through the direction perpendicular to the friction direction. From Figure 7b,d, wear marks are uniform and the wear mark surface is relatively regular. This reflects that the use of lubricant not only improves the wear scar morphology, but also reduces the wear amount. For condition 1 (720 °C/11.42 N/4.50 Hz), after using lubrication the specimen worn surface becomes smoother and more uniform than it is under dry conditions. By contrast, for condition 2 (650 °C/9.69 N/12.62 Hz), there are still many small peaks in the friction interface after using lubricant, but the depth of the wear marks is greatly reduced and more uniform than that under dry friction conditions. The reason for the occurrence of the small peak may be that lubricant particles are mounted in the lubrication film at the friction interface under the experimental conditions.

### 3.3. Chemical and Component Analysis of the Contact Interface

**Figure 8:**
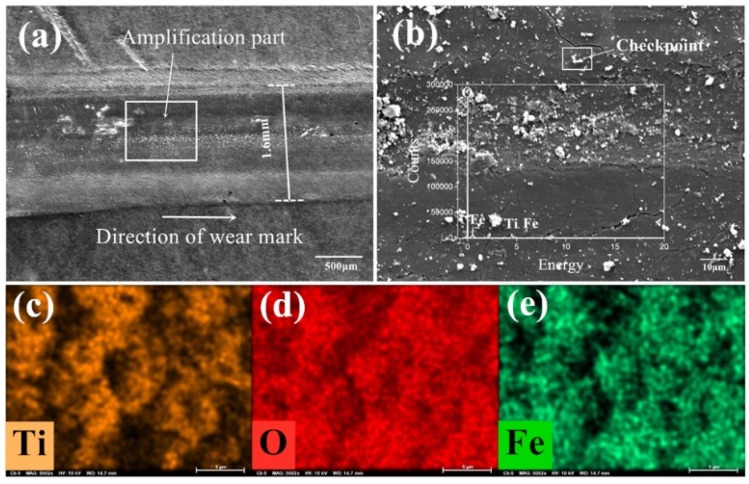
Detection image under experimental condition 1 (720 °C/11.42 N/4.50 Hz) without lubrication (**a**) the appearance of the wear marks; (**b**) the micromorphology of the wear marks at a magnification of 5000×; the element distribution map of (**c**) Ti, (**d**) O and (**e**) Fe on the detected surface of the wear mark.

Figure 8a–e is the SEM image and element distribution under the condition of 720 °C/11.42 N/4.50 Hz without lubrication. Figure 8a,b are the appearance of the wear marks in micromorphology with a magnification until 5000 times. Figure 8c–e are the distribution of elements on the detected area of wear marks. Ti, O and Fe elements can be found on the worn marks after the tribological test from the elemental distribution diagram and the peak position distribution of each element in Figure 8b. There are iron oxide and titanium oxide forms on the surface of the wear marks. The titanium oxide scale is covered on the wear scar, which prevent the direct contact of the friction pair and reduces the friction and wear to a certain extent. The iron oxide in some certain form can be considered as a kind of high-quality wear-resistant material or abrasive particles, and it is possible to improve or destroy the surface contact state.

From Figure 8a, the width of the wear mark is about 1.6 mm, which can be divided into a plastic deformation area and main worn area in the direction perpendicular to the wear mark. Both sides of the wear mark are plastic deformation areas located on both sides of the wear scar, while the main worn area is in the middle of the wear track. No obvious furrow and scratch can be seen, instead, a relatively shallow row of fishbone-like stripes can be found in the upper left corner of the wear mark. It is the crack-produced tearing area during the friction process. The O element is distributed over the whole interface, but the distribution intensity is quite different, which is due to the elements distribution of Ti and Fe in the forms of oxide with Ti^4+^ and Fe^3+^ ions. Part of the Ti element from the contact surface is replaced by the Fe element. The iron oxide may accelerate the deformation of titanium oxide scale. The distribution of Ti and Fe is approximately misaligned. However, in some regions the distribution of the two elements are overlapped. Under the proposed experimental condition, there is no uniform and protective covering film formed at the abrasion mark of the lower sample. Some iron oxide is pressed into the sample surface. The products from the tribological and thermal actions on the surface peel off and expose the base metal. Only some titanium and iron oxide are overlapped and covered on the surface of wear mark to perform as a protective film.

**Figure 9:**
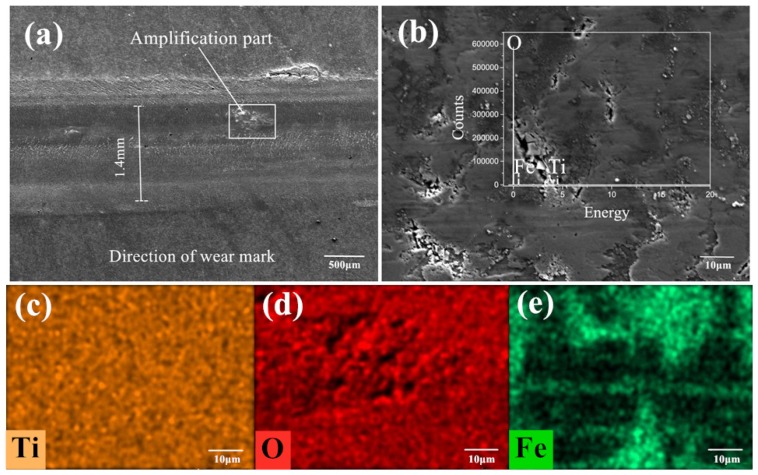
Detection image under experimental condition 2 (650 °C/9.69 N/12.62 Hz) without lubrication (**a**) the appearance of the wear marks; (**b**) the micromorphology of the wear marks at a magnification of 5000×; the element distribution map of (**c**) Ti, (**d**) O and (**e**) Fe on the detected surface of the wear mark.

Figure 9a–e is the SEM image and element distribution mapping detected under conditions of 650 °C/9.69 N/12.62 Hz. Similar to the results detected under condition 1 above, Ti, O and Fe elements have been also detected on the worn surface. In Figure 9a, the width of the wear mark is about 1.4 mm, which is similar to the result of condition 1 above. The wear mark area also presents both plastic deformation area and worn area. Several striations can be seen on the contact surface. Compared with the elements’ distribution in Figure 8, the Ti element covers a wider distribution range. Ti element is distributed in the whole detection area, while O element is weak in the detected area. The distribution of Fe locates on the plastic deformation area, while some Ti element is overlapped on these areas. This indicates the iron oxide is compressed in a thin film covered on the titanium substrate with less titanium oxide scale [38,39]. Meanwhile, the surface presents less oxide particle spread on the wear track with a compacted deformation area. In a lower rolling temperature, less oxide scale performs as a solid lubrication in the hot interfaces, which also contributes to a lower coefficient of friction.

**Figure 10:**
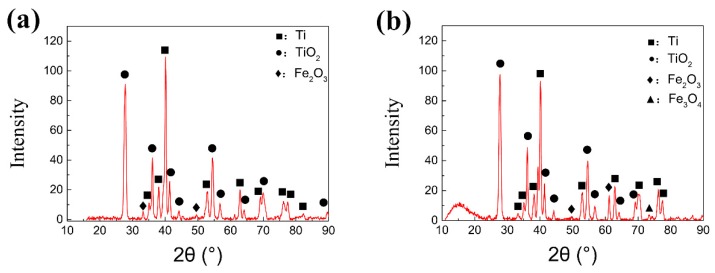
X-ray diffraction (XRD) analysis of the worn surface in dry friction under (**a**) Conditions 1 (720 °C/11.42 N/4.50 Hz) and (**b**) Conditions 2 (650 °C/9.69 N/12.62 Hz).

In order to further confirm the compound on the wear track, X-ray diffraction analysis has been performed on the worn surface, as shown in Figure 10. Oxides of titanium and iron can be found on the surface of the wear marks in both conditions 1 and 2 under dry condition. Clear diffraction peak of Fe_3_O_4_ can be only viewed in condition 2 at 650 °C. From the oxidation theory [40], the iron oxide scale above 600 °C is composed of FeO, Fe_3_O_4_ and Fe_2_O_3_ from the inner to the outer layer. Fe_3_O_4_ is the most compact layer with a good mechanical property compared with another two kinds of iron oxide. Only Fe_2_O_3_ was detected on the worn surface under the condition 1, which indicates that the thickness of the Fe_2_O_3_ layer at the outermost layer became thicker with increasing temperature. The brittle Fe_2_O_3_ scale peels off from the upper steel testing ball to form wear debris during the friction process. In condition 2, the thickness of Fe_2_O_3_ scale is thinner. In the friction process, the Fe_3_O_4_ peels off to the wear mark under the action of friction, and then forms a discontinuous protective film [41], which also causes a lower COF at 650 °C.

**Figure 11:**
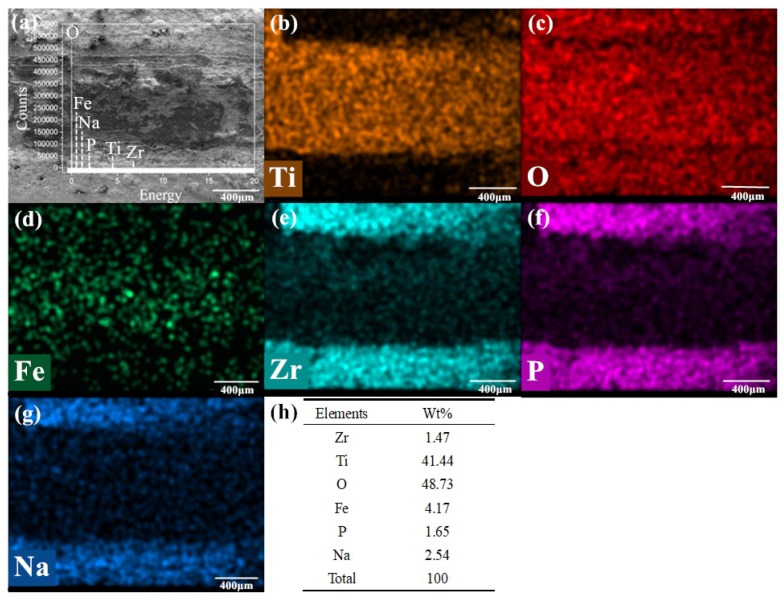
Detection image under experimental condition 1 (720 °C/11.42 N/4.50 Hz) with lubrication (**a**) the appearance of the wear marks; the element distribution map of (**b**) Ti, (**c**) O, (**d**) Fe, (**e**) Zr, (**f**) P and (**g**) Na on the detected surface of the wear mark; (**h**) elements by wt %.

Figure 11a–e is the SEM image and element distribution under the condition of 720 °C/11.42 N/4.50 Hz with mixed graphene-incorporating lubricant. Figure 11a are the appearance of the wear marks. Figure 11b–h are the distribution of elements and their mass percentages on the detected area of the wear marks. Ti, O, Fe, Zr, P and Na elements can be found on the worn marks after the tribological test from the elemental distribution diagram and the peak position distribution of each element in Figure 11a. There are some residual lubricating elements such as P, Na and Zr that can be found on the worn surface. But the surface is mainly covered by oxides of iron and titanium. Little C element can be found on the worn track. The O element is distributed over the whole interface. P, Na and Zr elements are mainly distributed both outside and near the edges of the wear mark. This means that most of the lubricants are extruded outside the wear mark during tribological process. The iron oxide scale locates mainly inside the wear marks, but there are still some iron oxide scales distribute outside the wear mark, which are the wear debris produced from the upper testing ball during friction. In the wear marks, the Zr element also presents similar distribution tendency with P and Na, which shows that ZrO_2_ nano particles possess some lubricating performance during hot tribological process [42,43]. The composition of graphene is not detected, which means that the graphene is exhausted at this temperature. It also shows that the lubricant composition on the wear mark surface is not covered by a continuous lubricant film. The lubricant film formed on the friction interface has been broken. At 720 °C, the friction-reduction and anti-wear components on the friction interface are mainly ZrO_2_ nano particles with a part of the phosphate lubricant composition.

**Figure 12:**
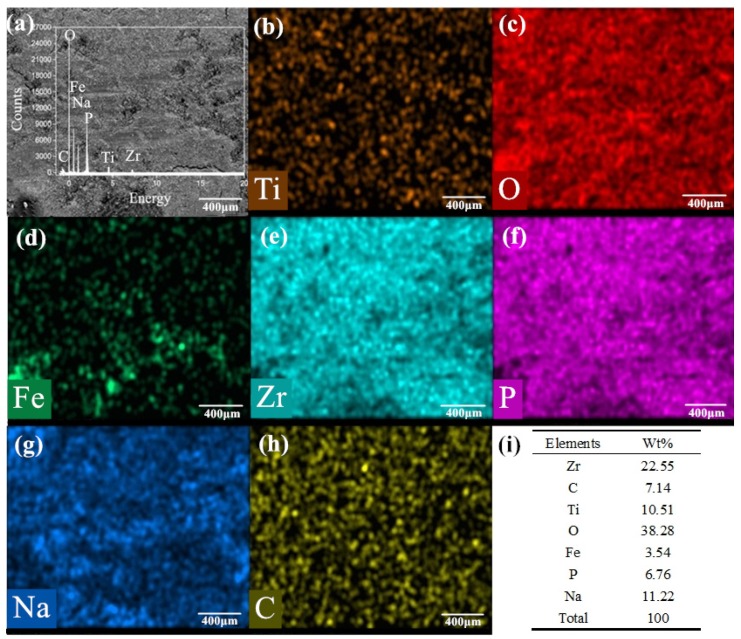
Detection image under experimental condition 2 (650 °C/9.69 N/12.62 Hz) with lubrication (**a**) the appearance of the wear marks; the element distribution map of (**b**) Ti, (**c**) O, (**d**) Fe, (**e**) Zr, (**f**) P (**g**)Na and (**h**) C on the detected surface of the wear mark; (**i**) elements by wt %.

Figure 12a–h is the SEM image, element distribution and their mass percentages under the condition of 650 °C/9.69 N/12.62 Hz with mixed graphene-incorporating lubricant. Compared with the test results at 720 °C, the lubrication film is uniform on the surface of the wear marks. It can be seen clearly that the elements of P, Na and Zr which is from the lubricating material are uniformly distributed on the worn surface. From the distribution mapping of the Ti element, the lubricating film isolates the base metal from the upper sample. The iron and oxygen elements are overlapped in the wear track, which indicates that iron oxide is formed during the friction process. The C element is detected in the elemental mapping. This shows the possibility for the existence of graphene in the wear track after the tribotest. It can also be found from the table in Figure 12h that the proportion of elements in lubricants is relatively obvious. It certifies that the lubricant composition on the surface of the wear marks still exists after 30 min of friction. Compared with the test results under experimental condition 1 with lubrication above, the lubrication film is uniform and continuous covered on the surface of the wear marks. Under the synergistic action of the lubricants, an effective barrier layer is formed on the surface of the friction pairs [44,45]. It effectively reduces the friction coefficient. The friction coefficient and wear rate are greatly reduced at the friction interface because of the excellent friction reducing materials at high temperatures.

**Figure 13:**
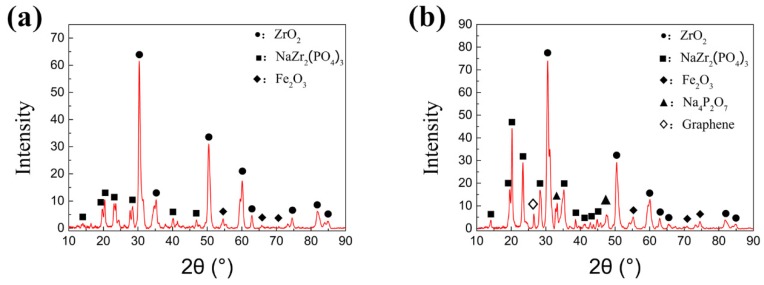
XRD analysis of the worn surface with lubricant under (**a**) Conditions 1 (720 °C/11.42 N/4.50 Hz) and (**b**) Conditions 2 (650 °C/9.69 N/12.62 Hz).

X-ray diffraction analysis was carried out for the phase composition on the worn surface of the specimens obtained under two experimental conditions with lubrication, as shown in Figure 13. A fine step size is set as 0.02° for the test accuracy. The film information can be obtained from the equipment although the basement may also be detected if some part of the film is too thin. It can be seen from Figure 13a that under the condition 1(720 °C/11.42 N/4.50 Hz), the lubricant component ZrO_2_ and the lubricant product NaZr_2_(PO_4_)_3_ are detected on the wear mark surface. This indicates that in the lubricant components chemical reactions occur at the wear mark interface. The phosphate melts at high temperature with increasing ion transfer rate and diffusion rate. When the phosphate melts, the phosphate and zirconia undergo atom rearrangement and electron transfer, so as to form sodium zirconium phosphate which is advanced for the metal forming process. However, the graphene is not detected in this condition, which may be due to the combustion loss of graphene itself at 720 °C.

From Figure 13b, it can be found that there are five identical lubricant product components including graphene from the wear mark interface at 650 °C. The sodium zirconate phosphate shows a unique skeleton structure with high thermal and chemical stability. The structure of NaZr_2_(PO_4_)_3_ also shows high isomorphic ability, which is conducive to combining the ions with different ionic radius, valence state and electronic shell structure. It is beneficial to form uniform and adhesion chemical adsorption film on the surface of the base metal. The phosphate not only provides the oxidation resistance, but also a foundation base for the connection of iron oxide particles, ZrO_2_ nano particles and graphene. Meanwhile, the graphene and nano particles are able to strengthen the tribofilm, which prompts higher wear resistance and friction reduction performance during tribological process. Owing to the excellent synergistic effect of the lubricating materials, a stable lubrication film with high bonding strength is formed in the friction process so as to greatly reduce the COF and wear volume.

**Figure 14:**
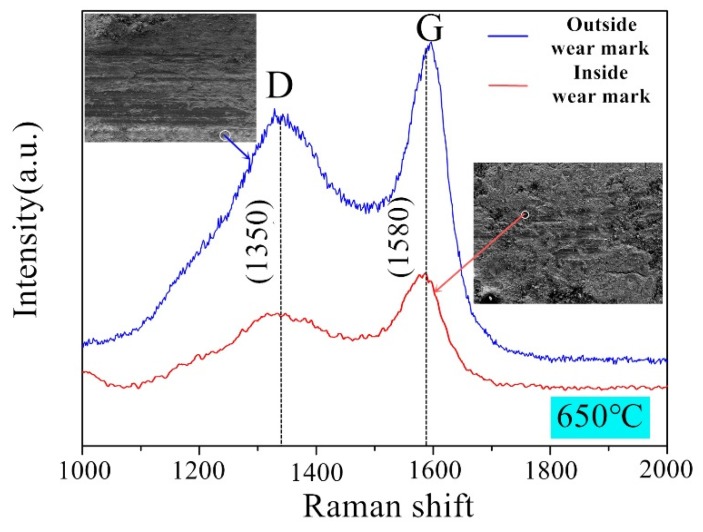
Raman results on internal and external of wear marks under Conditions 2 (650 °C/9.69 N/12.62 Hz) with mixed graphene-incorporating lubricant.

The Raman spectra of the worn surface of the sample have been measured at targeted points to confirm the existence of graphene under condition 2 with lubrication. The Raman results are obtained by detecting both inside and outside of the wear marks, as shown in Figure 14. It is generally believed that the characteristic peak of graphene exists the D peak at 1350 cm^−1^ and the G peak at 1580 cm^−1^ [46,47,48]. The diffraction peaks have been detected at 1350 cm^−1^ and 1580 cm^−1^ in both curves with a similar tendency. The results of the two detection sites confirm the existence of graphene. Although most of the graphene can be found in the wear marks which act as an excellent lubricating material, there are still some graphene may be extruded from the wear marks to the external area or consumed at high temperature.

### 3.4. Chemical- and Mechanical-Induced Lubrication Mechanism

**Figure 15:**
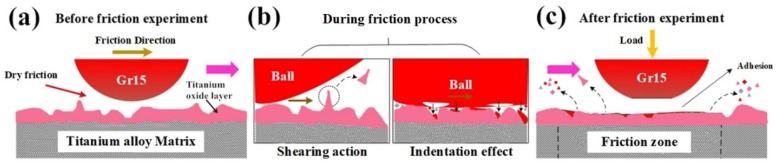
Schematic diagram of tribological mechanism under dry friction condition (**a**) before (**b**) during and (**c**) after friction process.

Figure 15 below is a schematic diagram of the tribological mechanism at the tribo-interface under dry friction conditions. Before the friction experiment, the surface micro morphology of the upper and lower samples are rough due to the oxidation of both steel ball and titanium alloy disc. During the contact process, at the contact interface there mainly occur two processes as shown in Figure 15b. One process is the convex part of the surface on the lower sample is cut under the action of the shear force generated by the sliding with upper steel sample. The cut convex material forms the wear debris, which is peeled off from the contact interfaces. Another process is the indentation and adhesion phenomenon under high temperature and high pressure condition. The hard material (steel ball) of the upper sample forms a bonding layer of oxide steel on the surface. The soft materials (titanium alloy disc) is under the action of high temperature and pressure with the contact pair. Part of the upper iron oxide scale is compressed into the titanium oxide layer of the lower sample or adhered to the lower sample surface. In this stage, the rough points on the titanium oxide layer are mainly attrited by the shear force from upper test ball, so the wear of both upper steel ball and lower titanium disc are serious and the friction coefficient increases accordingly. Under the action of friction, the movement of debris formed during friction process between contact pairs leads to a plough groove, which is a typical feature of abrasive wear. Some parts of the titanium oxide layer are deformed during the friction process with the iron oxide particles. After the friction experimental process, a discontinuous and irregular oxide layer forms on the titanium alloy surface of the sample, which also accelerates the friction and wear process, as shown in Figure 15c.

On the consideration of the hot-rolling process of titanium alloy sheets, the absence of cooling water and lubrication during the hot-rolling process goes against the surface quality of titanium alloy, as well as the life of the work rolls. The tribological tests under dry friction conditions indicate the main wear types are adhesive and abrasive wear. During the hot-rolling process, the deformation speed may reach 5 m/s. The steel work roll material is much harder than the titanium alloy sheets. Even small wear particles at the interfaces may lead to serious scratches on the sheet surface, which is not qualified for the production requirement.

**Figure 16:**
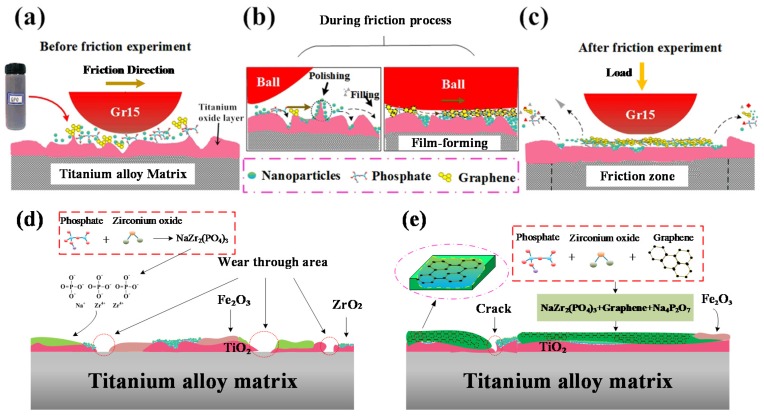
Schematic diagram of tribological processes under lubricated conditions (**a**) before (**b**) during and (**c**) after friction process and the chemical and mechanical induced lubrication mechanism at (**d**) 720 °C and (**e**) 650 °C.

Figure 16 shows a schematic diagram of the tribological process and its chemical- and mechanical-induced lubrication mechanism after conducting mixed graphene-incorporating lubricant. From the previous work [49], this kind of modified layer normally is about 100–950 nm in thickness from 600–800 °C. The thickness increases with the increased temperatures. When the temperature reaches 700 °C, the layer thickness increases sharply due to the intense chemical reactions on the tribo-interfaces at elevated temperatures. From Figure 16a, the contact surface is uneven before applying the proposed mixed graphene-incorporating lubricant due to the metal oxidation at high temperatures. When the lubricant is added, the lubricant components which are mixed in a disorderly way start a molding process under the effect of high temperature and high pressure. When the mixed lubricant enters into the wear mark, the water vapor firstly boils and vaporizes. The phosphate, ZrO_2_ nano particles and graphene are left between the contact interfaces. During the vaporization, the mixed lubricant components are uniformly mixed. Molten phosphate flows over the whole wear tracks and it can be firmly attached to the contact surface. Under the action of relative sliding and high-temperature conditions, the phosphate between the contact steel-titanium pairs acts as the carrier and forms a phosphate-graphene lubrication film. This lubrication film prevents direct contact between friction pairs. The presence of graphene plays the role of a skeleton which can be used for strengthening the molten phosphate film. It improves the strength of the lubricating film so as to avoid cracks after the friction process. The graphene which is embedded into the phosphate film can be effectively applied as a lubricating material with less combustion loss at such a high temperature. Nano ZrO_2_ plays different roles because of their size advantages. Some nanoparticles play a role of bearing pressure on the contact surface. Some parts enter the voids and cracks of the interface during the friction process to make the contact surface flatter. The ZrO_2_ nano particles also work on repairing surface defects. This reflects the self-repairing function of nano particles. Some nanoparticles present a polishing function to grind off the protruding parts of the surface, which reduce the roughness of the contact surface. This can be considered a mechanical-induced lubrication mechanism of the mixed graphene-incorporating lubricant. Therefore, the COF of the tribological surface is greatly reduced and the wear volume is also decreased under the synergistic action of the proposed mixed graphene-incorporating lubricant.

Figure 16d,e shows the chemical- and mechanical-induced lubrication mechanism under two experimental conditions (similar conditions from the hot rolling of titanium at stand 1 and 6 on finishing mills) respectively. Under condition 1 (720 °C/11.42 N/4.50 Hz), it can be seen from Figure 16d that the formation of the lubrication interface is mainly composed of TiO_2_ oxide layer and fragmented lubrication layer. During the tribological process, the high temperature environment causes the formation of a core-rim structure of the TiC particles [50]. The TiC particle presents a much higher hardness compared to the matrix alloy. This indicates the TiC particle enhances the resistance to mechanical damage and wear [51]. The Fe-TiC composite may also be produced under this condition with iron oxide particles from the ball. The wear properties of the Fe-TiC composite under different conditions have been described as hard carbide particles for the increase of wear resistance [52]. After applying lubricant, phosphate reacts chemically with zirconia to form NaZr_2_(PO_4_)_3_ lubrication component. The sodium zirconate phosphate presents a high isomorphic ability owing to its unique skeleton structure with high thermal and chemical stability. It is chemically welcomed in order to combine the ions with different ionic radius, valence state and electronic shell structure. The new generated lubrication film shows well wear reduction and friction resistance performance at the first 15 min. However, the COF tends to be increased after a certain friction process. It indicates the lubrication life is reasonable but still need to be improved during the hot rolling of titanium alloy at stand 1 of a hot finishing mill. In some worn areas, the lubricating layer and oxide layer are worn through to expose the base metal. There are also iron oxide scales in the lubricating films. This is the reason why the COF increases with the friction time. Under condition 2 (650 °C/9.69 N/12.62 Hz), owing to the existence of graphene during the friction process, the lubrication film is continuous with both original lubrication materials and tribo-chemical products. The graphene improves the strength of the lubrication film, which extends the life of the tribofilm. The chemical- and mechanical-induced lubrication film shows excellent lubricating performance due to the synergistic lubrication mechanism of the graphene, phosphate and ZrO_2_ nano particles at high temperatures with load.

From the industrial production experience of titanium alloy sheets by hot rolling, the surface defects normally happen at the exit of the stand 1 and stand 6 of a hot-finishing mill. Under both hot rolling conditions, the mixed graphene-incorporating lubricant shows good lubrication characteristics, especially in the condition of stand 6 at 650 °C with a synergistic action of graphene and other lubrication materials. It provides the possibility for the proposed mixed graphene-incorporating lubricant to be applied for the hot rolling of titanium alloy sheets so as to improve the surface quality and roll life. These results may also provide guidelines for optimizing the lubrication performance with other synergistic lubrication mechanism which are subjected to the hot metal forming.

## 4. Conclusions

In this study, the chemical- and mechanical-induced lubrication mechanism of mixed graphene-incorporating lubricant has been investigated under the condition of hot rolling of titanium alloy sheets. Under dry friction condition, the average COF is 0.575 and 0.559, respectively according to the titanium alloy rolling parameters of stand 1 and stand 6 in the hot finishing mill. After applying the proposed mixed graphene-incorporating lubrication, the average friction coefficient decreases by 30.27% and 35.22%, respectively. Compared with the samples obtained from dry friction, the lubricated surface is smoother and the surface defects are greatly reduced. The surface cracks, holes, and peeling debris are significantly decreased. The plough on the worn surface is no longer obvious. A uniform coverage of lubrication film can be found. The protective film forms an isolation layer at the friction interface, which prevents uneven contact between friction pairs. It proves that the proposed lubricant plays a critical role in reducing the friction coefficient during the hot rolling of titanium alloy sheets.

The typical wear features are abrasive wear and adhesive wear under dry friction during the rolling process of a titanium sheet. While under the action of relative sliding movement and high temperature with lubrication conditions, the phosphate between the contact steel-titanium pairs acts as the carrier and forms a phosphate-graphene lubrication film. Phosphate also reacts chemically with zirconia to form a NaZr_2_(PO_4_)_3_ lubrication component. The sodium zirconate phosphate presents high isomorphic ability owing to its unique skeleton structure with high thermal and chemical stability. The presence of graphene plays a role of skeleton which can be used for strengthening the molten phosphate film. The graphene which is embedded into the phosphate film can be effectively applied as a lubricating material with less combustion loss at such a high temperature. Nano ZrO_2_ particles play a number of roles because of their nano size advantages. Due to the synergistic lubrication effect of the graphene, ZrO_2_ nano particles and phosphate with the proposed lubricant, a chemical- and mechanical-induced lubrication mechanism for the hot rolling of titanium sheets has been proposed with the mixed graphene-incorporating lubricant. The proposed lubricant exhibits promising prospects to be a hot-rolling lubricant candidate for the production of titanium alloy sheets.

## Figures and Tables

**Table 1:** Lubricant composition (in wt %).

Phosphate	ZrO_2_	PVP	Graphene	Balanced Water
9	4	1	0.05	allowance

**Table 2:** Chemical composition (in wt %) of Ti-0.3Mo-0.8Ni Alloy.

Ti	O	C	H	N	Fe	Mo	Ni
Balance	≤0.18	≤0.08	≤0.015	≤0.03	≤0.30	0.2–0.4	0.6–0.9

**Table 3:** Chemical composition (in wt %) of GCr15 steel balls.

C	Cr	Mn	Si	S	P
0.95–1.05	1.30–1.60	0.20–0.40	0.15–0.35	≤0.02	≤0.027

**Table 4:** Experimental conditions.

Condition	Temperature/°C	Load/N	Frequency/Hz	Time/s
1	720	11.42	4.50	1800
2	650	9.69	12.62	1800

**Table 5:** Average friction coefficients under various experimental conditions.

Condition	Dry	Lubricated
1	0.575	0.401
2	0.559	0.362
